# Supplementary material for: Modulating Kinetics of the Amyloid-Like Aggregation of S. aureus Phenol-Soluble Modulins by Changes in pH
Source: Microorganisms. 2021 Jan 7;9(1):117. doi: 10.3390/microorganisms9010117 (PMC7825627; doi:10.3390/microorganisms9010117)
Supplement: Supplementary file 1 [file microorganisms-09-00117-s001.pdf]

Supplementary figures

# Modulating kinetics of amyloid-like aggregation of *S. aureus* phenol soluble modulins by changes in pH

Masihuz Zaman and Maria Andreasen

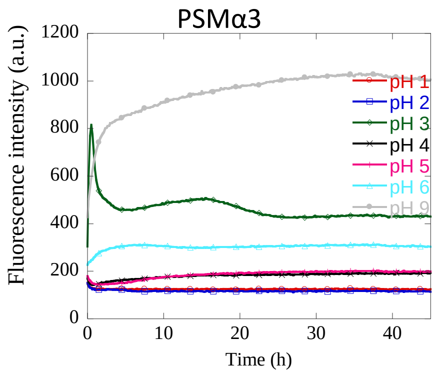


**Figure S1: Aggregation of PSMα3 at 37 °C**. Experimental kinetics of PSMα3 aggregation at 37°C every 10 min under quiescent conditions for single monomeric concentration (0.5 mg/ml) at pH 1-7.

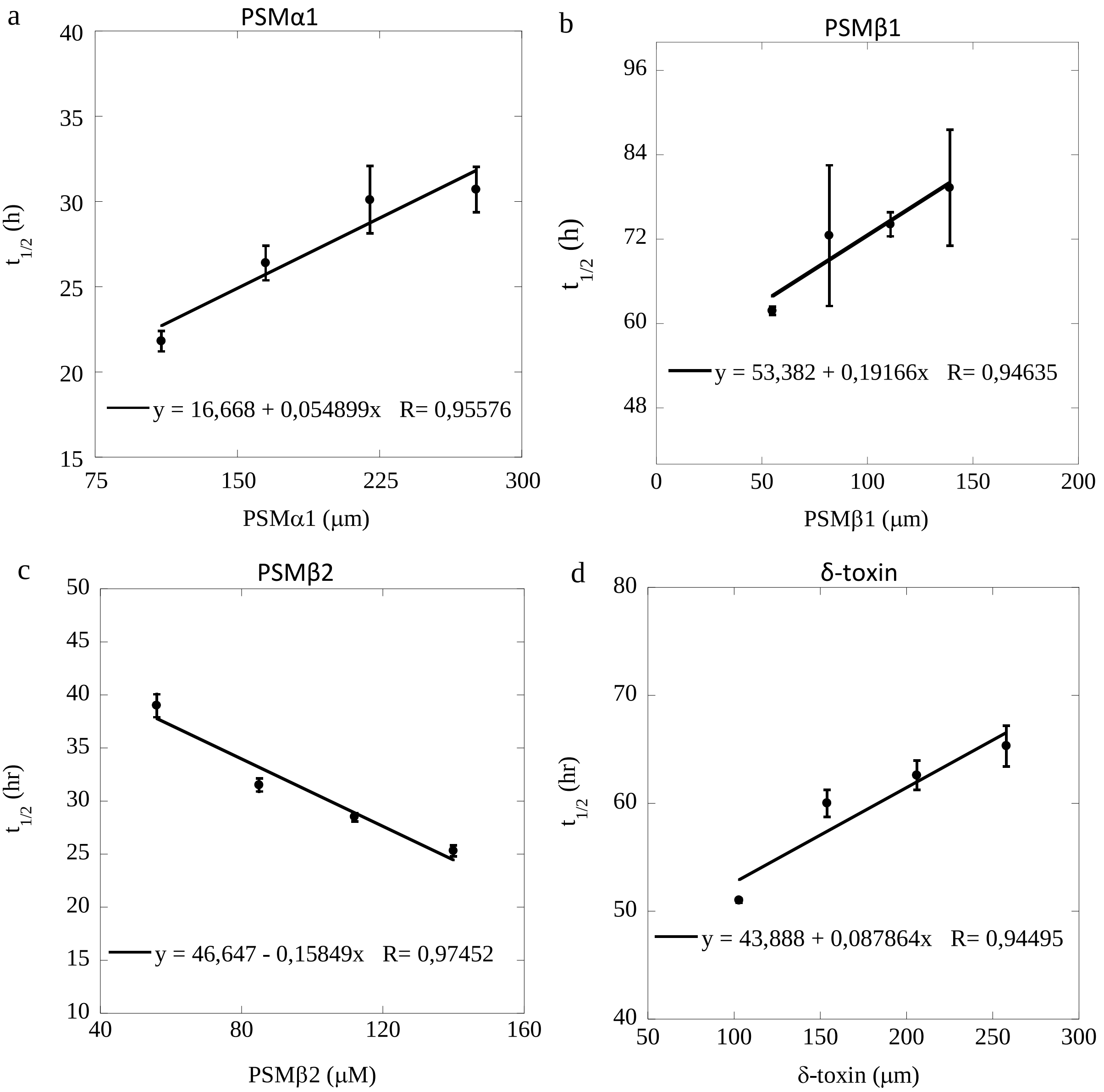


**Figure S2: Half-time plots of PSMs**. Dependence of $t_{1/2}$ values on the various concentrations of PSMs peptides used in the aggregation assays. Four different concentrations for each peptide were studied at fixed pH. In all figures, the error bars correspond to the standard deviations of kinetic parameters determined for a single experiment in which each peptide concentration was run in triplicates. (a) Dependence of the $t_{1/2}$ on the concentration of PSMα1. (b) Dependence of the $t_{1/2}$ on the concentration of PSMβ1. (c) Dependence of the $t_{1/2}$ on the concentration of PSMβ2. (d) Dependence of the $t_{1/2}$ on the concentration of δ-toxin.

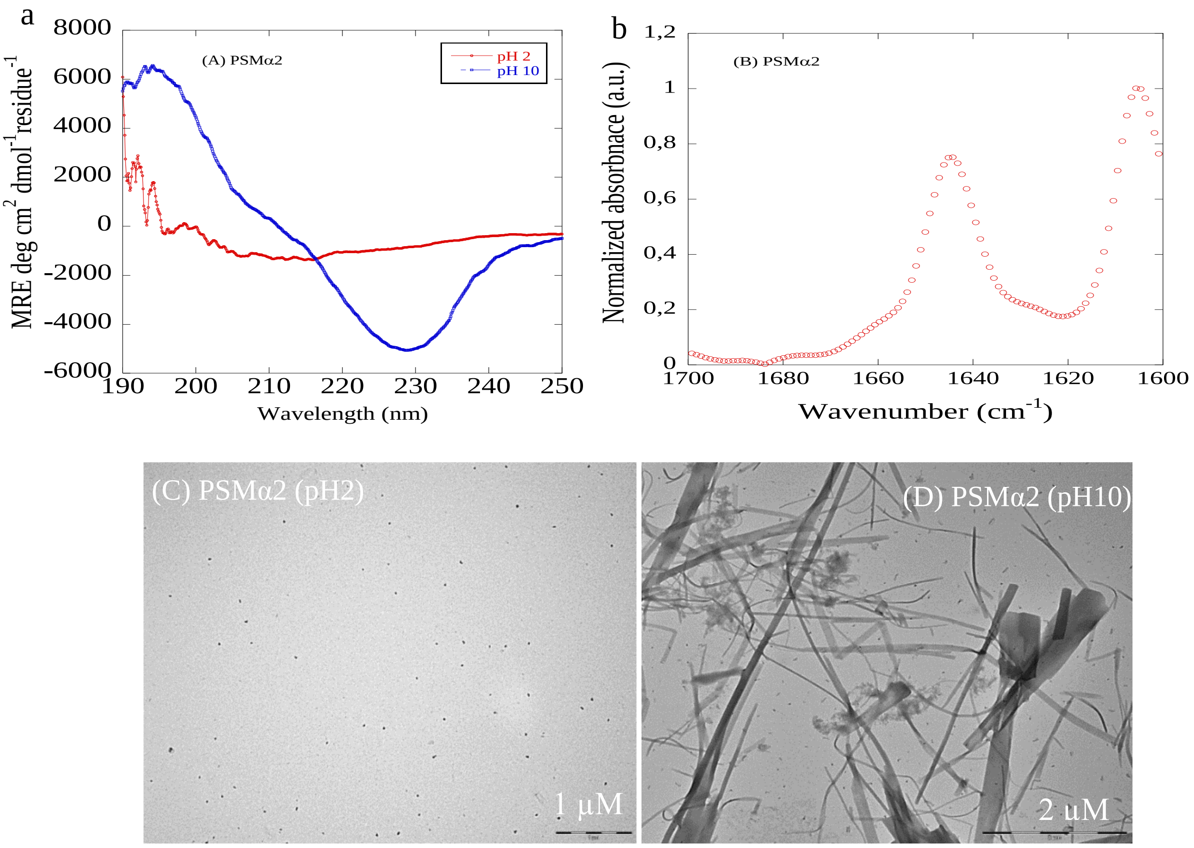


**Figure S3: Structural and morphological characteristics of PSMα2 at 37°C**. (a) Far-UV CD spectra of PSMα2 at pH 2 and pH 10. (b) Attenuated total internal reflection Fourier transform infrared (ATR-FTIR) spectroscopy of the amide I' region (1600-1700 cm-1) of fibrils of PSMα2 at pH 10. (c) Transmission electron microscopy of PFSMα2 fibril at acidic (pH2) conditions. (d) Transmission electron microscopy of PSMα2 fibril at basic (pH10) condition.

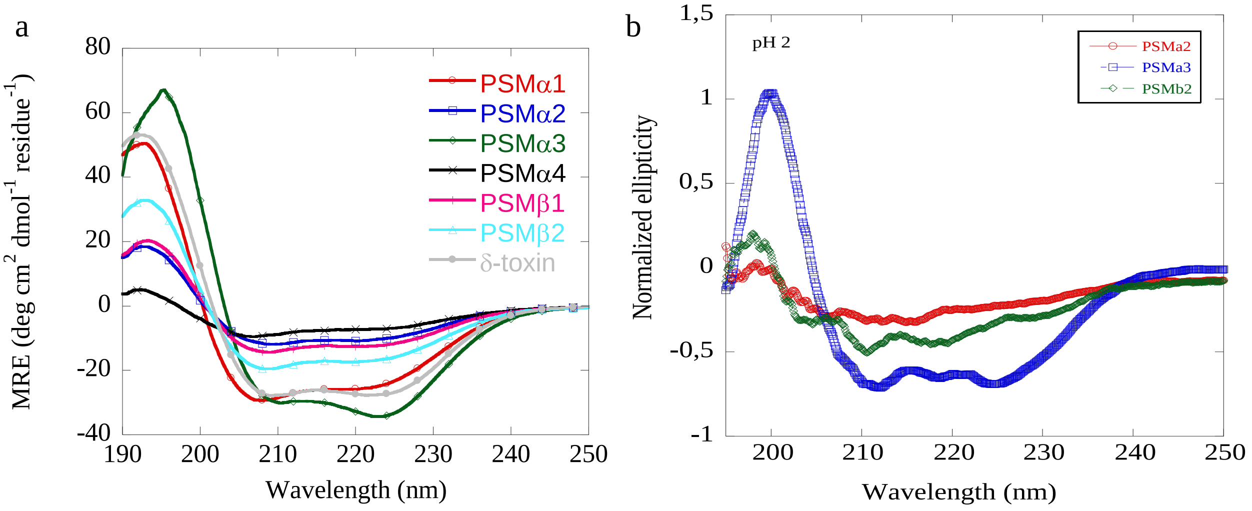


**Figure S4: Far UV-CD structural characterization of PSM peptides**. (a) Far UV-CD spectra of monomeric PSM peptides. (b) Far UV-CD spectra of PSMα2, PSMα3 and PSMβ2 at pH 2.

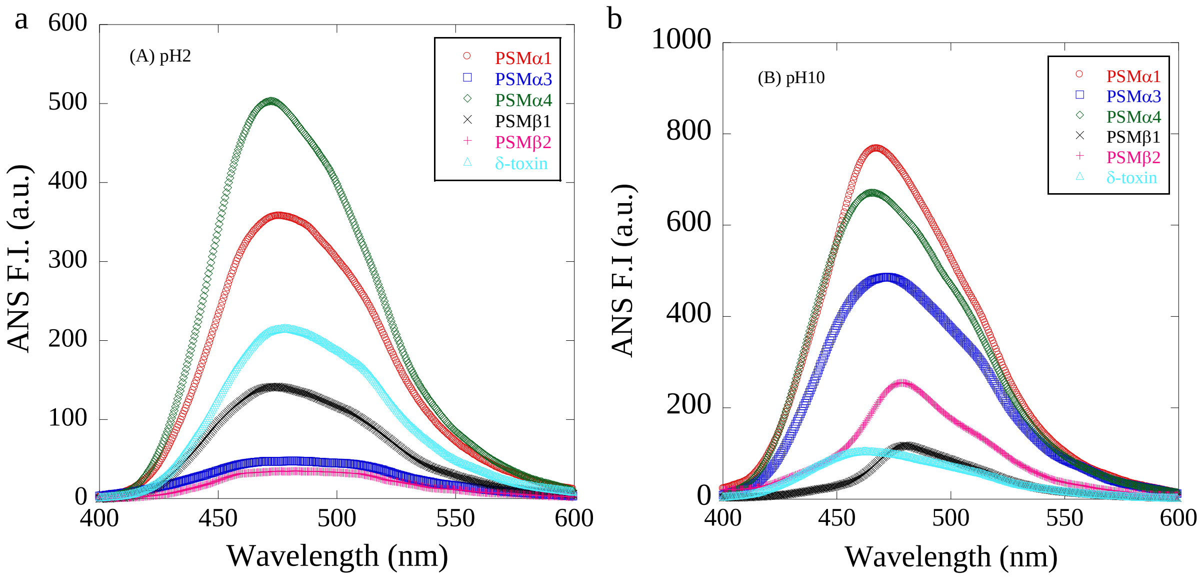


**Figure S5: ANS fluorescence emission spectra of PSMs fibrils**. (a) ANS fluorescence emission spectra of PSMs fibrils at acidic (pH2) conditions. (b) ANS fluorescence emission spectra of PSMs fibrils basic (pH10) conditions.

**Table S1: Charge and p$K_a$ of PSM peptides.** Charge and p$K_a$ value of different peptides at numerous pH calculated by protein calculator v3.4.

| pH | Charge on Peptides | | | | | | |
|---|---|---|---|---|---|---|---|
| | PSMα1 | PSMα2 | PSMα3 | PSMα4 | PSMβ1 | PSMβ2 | δ-toxin |
| | pKa= 9.72 | pKa= 10.00 | pKa= 9.53 | pKa= 9.72 | pKa= 4.89 | pKa= 5.69 | pKa= 8.69 |
| 1.00 | 4.0 | 5.0 | 5.0 | 4.0 | 4.0 | 4.0 | 5.0 |
| 2.00 | 3.9 | 4.9 | 4.9 | 3.9 | 3.9 | 3.9 | 4.9 |
| 3.00 | 3.5 | 4.5 | 4.5 | 3.5 | 3.4 | 3.4 | 4.4 |
| 4.00 | 2.8 | 3.8 | 3.5 | 2.8 | 2.0 | 2.3 | 3.3 |
| 5.00 | 2.2 | 3.2 | 2.4 | 2.2 | -0.2 | 0.6 | 1.6 |
| 6.00 | 2.0 | 3.0 | 2.0 | 2.0 | -0.9 | -0.2 | 1.1 |
| 7.00 | 1.9 | 2.9 | 1.9 | 1.9 | -1.1 | -0.8 | 0.9 |
| 8.00 | 1.5 | 2.5 | 1.5 | 1.5 | -1.5 | -1.5 | 0.5 |
| 9.00 | 0.8 | 1.7 | 0.7 | 0.8 | -2.2 | -2.1 | -0.3 |
| 10.00 | -0.5 | 0.0 | -1.0 | -0.5 | -3.5 | -3.0 | -2.0 |
| 11.00 | -1.7 | -1.6 | -2.6 | -1.7 | -4.7 | -3.8 | -3.6 |
| 12.00 | -2.0 | -2.0 | -3.0 | -2.0 | -5.0 | -4.0 | -4.0 |

**Table S2:** Kinetic parameters of PSM aggregation. Summary of the averages and standard deviations of the kinetic parameters obtained from the ThT assays for different PSMs at various pH Values.

| Peptide | Peptide concentration (mg/ml) | $t_{1/2}$ (hr) | *lag time (hr)* |
|---|---|---|---|
| PSMα1 (pH2) | 0.25 mg/ml | 21.8 ± 0.60 | 17.9 ± 0.62 |
| | 0.37 mg/ml | 26.4 ± 1.02 | 18.3 ± 0.69 |
| | 0.50 mg/ml | 30.1 ± 1.98 | 18.9 ± 0.30 |
| | 0.62 mg/ml | 30.7 ± 1.34 | 20.8 ± 1.93 |
| PSMβ1 (pH10) | 0.25 mg/ml | 61.8 ± 0.60 | 52.8 ± 0.72 |
| | 0.37 mg/ml | 72.0 ± 10.0 | 53.1 ± 8.39 |
| | 0.50 mg/ml | 74.0 ± 1.73 | 44.7 ± 0.22 |
| | 0.62 mg/ml | 80.0 ± 8.25 | 42.0 ± 0.65 |
| PSMβ2 (pH10) | 0.25 mg/ml | 39.0 ± 1.08 | 34.5 ± 0.71 |
| | 0.37 mg/ml | 31.5 ± 0.62 | 27.5 ± 0.74 |
| | 0.50 mg/ml | 28.5 ± 0.40 | 22.4 ± 0.43 |
| | 0.62 mg/ml | 25.3 ± 0.50 | 17.9 ± 0.86 |
| δ-toxin (pH2) | 0.30 mg/ml | 51.0 ± 0.20 | 35.2 ± 0.32 |
| | 0.45 mg/ml | 60.0 ± 1.27 | 42.6 ± 0.77 |
| | 0.60 mg/ml | 62.6 ± 1.34 | 46.5 ± 0.26 |
| | 0.75 mg/ml | 65.3 ± 1.87 | 46.9 ± 1.09 |

**Table S3:** FTIR spectral deconvolution. The percentage contribution of various structural components is given for the aggregated form of all seven peptides at acidic (pH2) and basic (pH10) conditions.

| Peptide | pH 2 | | | | | pH 10 | | | | |
|---|---|---|---|---|---|---|---|---|---|---|
| | Peak position | % β-sheet | % β-turn | %-α helix | % r-coil | Peak position | % β-sheet | % β-turn | %-α helix | % r-coil |
| PSMα1 | 1625, 1661 | 71.6 | - | 28.6 | - | 1626,1653, 1671 | 47.2 | 9.0 | 29.4 | - |
| PSMα2 | - | - | - | - | - | 1604,1628, 1644,1660 | 2.67 | 1.45 | - | 40.5 |
| PSMα3 | 1638,1656 | 36.1 | - | 34.8 | - | 1654 | - | - | 81.1 | - |
| PSMα4 | 1628,1655, 1695 | 56.7 | 19.6 | 23.7 | - | 1626, 1648 | 35.1 | - | - | 43.2 |
| PSMβ1 | 1624,1664 | 51.1 | 29.5 | - | - | 1629,1648, 1666 | 46.1 | 17.0 | - | 11.2 |
| PSMβ2 | - | - | - | - | - | 1625,1645, 1665 | 42.2 | 17.1 | - | 14.6 |
| δ-toxin | 1625,1669 | 79.9 | 20.1 | - | - | 1628,1645 | 3.2 | - | - | 39.6 |
